# “Warning: ultra-processed”: an online experiment examining the impact of ultra-processed warning labels on consumers’ product perceptions and behavioral intentions

**DOI:** 10.1186/s12966-024-01664-w

**Published:** 2024-10-09

**Authors:** Aline D’Angelo Campos, Shu Wen Ng, Ana Clara Duran, Neha Khandpur, Lindsey Smith Taillie, Fernanda O. Christon, Marissa G. Hall

**Affiliations:** 1 Department of Health Behavior, Gillings School of Global Public Health, University of North Carolina at Chapel Hill, Chapel Hill, NC USA; 2 Carolina Population Center, University of North Carolina at Chapel Hill, Chapel Hill, NC USA; 3 Department of Nutrition, Gillings School of Global Public Health, University of North Carolina at Chapel Hill, Chapel Hill, NC USA; 4 Center for Food Studies and Research (NEPA), University of Campinas, Campinas, SP Brazil; 5 Division of Human Nutrition and Health, Wageningen University, Wageningen, Netherlands; 6 Center for Epidemiological Studies in Health and Nutrition (NUPENS), School of Public Health, University of São Paulo, São Paulo, SP Brazil; 7 Department of Nutrition, Harvard T.H. Chan School of Public Health, Boston, MA USA; 8 Lineberger Comprehensive Cancer Center, University of North Carolina at Chapel Hill, Chapel Hill, NC USA

**Keywords:** Ultra-processed foods, Front-of-package labeling, Front-of-pack labeling, Warning labels, Food labeling

## Abstract

**Background:**

Nutrient content and degree of processing are complementary but distinct concepts, and a growing body of evidence shows that ultra-processed foods (UPFs) can have detrimental health effects independently from nutrient content. 10 + countries currently mandate front-of-package labels (FOPL) to inform consumers when products are high in added sugars, saturated fat, and/or sodium. Public health advocates have been calling for the addition of ultra-processed warning labels to these FOPLs, but the extent to which consumers would understand and be influenced by such labels remains unknown. We examined whether the addition of ultra-processed warning labels to existing nutrient warning labels could influence consumers’ product perceptions and purchase intentions.

**Methods:**

In 2023, a sample of adults in Brazil (*n* = 1,004) answered an open-ended question about the meaning of the term “ultra-processed,” followed by an online experiment where they saw four ultra-processed products carrying warning labels. Participants were randomly assigned to view either only nutrient warning labels or nutrient plus ultra-processed warning labels. Participants then answered questions about their intentions to purchase the products, product perceptions, and perceived label effectiveness.

**Results:**

Most participants (69%) exhibited a moderate understanding of the term “ultra-processed” prior to the experiment. The addition of an ultra-processed warning label led to a higher share of participants who correctly identified the products as UPFs compared to nutrient warning labels alone (Cohen’s d = 0.16, *p* = 0.02). However, the addition of the ultra-processed warning label did not significantly influence purchase intentions, product healthfulness perceptions, or perceived label effectiveness compared to nutrient warning labels alone (all *p* > 0.05). In exploratory analyses, demographic characteristics and prior understanding of the concept of UPF did not moderate the effect of ultra-processed warning labels.

**Conclusions:**

Ultra-processed warning labels may help consumers better identify UPFs, although they do not seem to influence behavioral intentions and product perceptions beyond the influence already exerted by nutrient warning labels. Future research should examine how ultra-processed warning labels would work for products that do and do not require nutrient warnings, as well as examine the benefits of labeling approaches that signal the health effects of UPFs.

**Trial registration:**

ClinicalTrials.gov, NCT05842460. Prospectively registered March 15th, 2023.

**Supplementary Information:**

The online version contains supplementary material available at 10.1186/s12966-024-01664-w.

## Introduction

Ultra-processed foods (UPFs) consist of industrial formulations that contain little or no whole food ingredients, are assembled using intense processing methods (including physical, chemical and biological processing), and contain synthetic additives such as flavorings, colorings, aromas, and emulsifiers [1]. A substantive body of epidemiological evidence shows an association between consumption of UPFs and a range of adverse health outcomes [2–10], with especially strong evidence linking higher UPF intake to increased risks of cardiovascular disease-related mortality, mental disorders, and type 2 diabetes [11]. While many UPFs are high in nutrients of public health concern (e.g., added sugars, saturated fats, sodium) and low in more beneficial nutrients (e.g., fiber, vitamins, minerals, protein) [12–16], nutrient content and degree of processing represent different product dimensions. Public health interventions such as food labeling have traditionally focused on foods’ nutrient content, but recognition of the additional importance of degree of processing for dietary patterns has been growing [17] as epidemiological evidence points to UPFs’ nutrient content only partly explaining their associations with adverse health outcomes [18–24].

Brazil has in many ways been at the forefront of public health efforts targeting UPF consumption. The Nova system, which was developed in Brazil, divides foods into four categories based on their degree of processing (i.e., 1 = unprocessed or minimally processed, 2 = processed culinary ingredients, 3 = processed foods, and 4 = ultra-processed foods) and provides the most widely accepted definition of UPFs globally [25]. Nova has had a large influence on Brazil’s general public health discourse, which culminated in the Brazilian Ministry of Health using Nova as the basis for its Dietary Guidelines for the Brazilian Population, published in 2015 [26]. The Guidelines recommend that Brazilians make unprocessed or minimally processed foods the basis of their diets, use culinary ingredients in small amounts to create culinary preparations, limit processed foods, and avoid UPFs [26]. Despite these efforts, UPF consumption remains a concern; as of 2018, 19.7% of the calories consumed in Brazil were estimated to come from UPFs, which represented a 5.5% increase from 2008 [27]; and as of 2022, around 10% of premature deaths among Brazilian adults were attributed to UPF consumption [28]. Importantly, recent increases in UPF consumption have been greater among individuals with the lowest income and education levels, individuals who identify as Black, *pardo* (i.e., “brown” or mixed-race) or Indigenous, and in the lowest-income regions of the country (i.e., North and Northeast) [27].

In 2022, in a parallel effort to improve population-level diet quality, Brazil joined the now more than 10 countries in the Americas that mandate interpretive nutritional front-of-package labeling (FOPL) for foods and non-alcoholic beverages. Interpretive FOPLs provide nutritional information through visual cues (symbols and graphics) to aid consumers’ interpretation of such information [29]. The World Health Organization recommends FOPLs for their wide reach and low cost [30], and studies show that FOPLs help consumers identify unhealthier foods, discourage their purchase, and encourage the food industry to reformulate many such products [29, 31]. Brazil’s FOPL scheme, similarly to other countries in the Americas, is composed of warning labels applied to products that exceed added sugar, saturated fat, and sodium thresholds set by the Brazilian Health Regulatory Agency (ANVISA). Although not all existing FOPL schemes use this warning label model, all convey information exclusively about products’ nutrient content [32]. However, experts and global advocacy organizations have proposed that information about products’ degree of processing also be incorporated into FOPLs [33]. In the Brazilian context, ultra-processed warning labels could enhance coherence between the country’s Dietary Guidelines and its FOPL scheme.

To date, the benefits of adding ultra-processed labels to existing nutrient labels remain unknown. Previous studies with samples from the United States and France suggest that different types of ultra-processed labels (i.e., an octagonal ultra-processed warning label and an ultra-processed banner as part of a summary nutritional label) could help consumers better identify UPFs, decrease perceived healthfulness of UPFs, and discourage consumption [34, 35]. However, in both studies, ultra-processed labels were only tested in comparison to control conditions (i.e., no labels or neutral labels). No research has yet examined whether there would be any benefits to changing the status quo in countries that already mandate FOPLs by adding ultra-processed labels to existing nutrient labels.

This study aimed to assess the impact of adding an ultra-processed warning label to nutrient warning labels on a sample of Brazilian consumers’ product perceptions and behavioral intentions. We hypothesized that participants who saw combined ultra-processed and nutrient warning labels would be more likely to identify all products presented as ultraprocessed and would report lower intentions to purchase the product, lower perceived product healthfulness, and higher perceived message effectiveness than participants who saw only nutrient warning labels. We also explored consumers’ prior understanding of the term “ultra-processed” to contextualize experimental results.

## Methods

### Participants

In June 2023, we recruited an online convenience sample using the survey research platform Cint as part of a parent study examining the impact of the use of different nutrient profile models in Brazil’s FOPL system. Participants were eligible if they were 18 years or older, resided in Brazil, and were responsible for at least 50% of their household’s food purchases. The panel company used purposive sampling to obtain a sample whose age, gender, and regional distribution was comparable to that of the Brazilian population. The study received approval from the University of North Carolina at Chapel Hill’s Institutional Review Board (#21-2129) and the University of Campinas Ethics in Research Committee. The study design, measures, hypotheses, and analytic plan were registered prior to data collection on ClinicalTrials.gov (NCT #05842460).

### Procedures

**Fig. 1:**
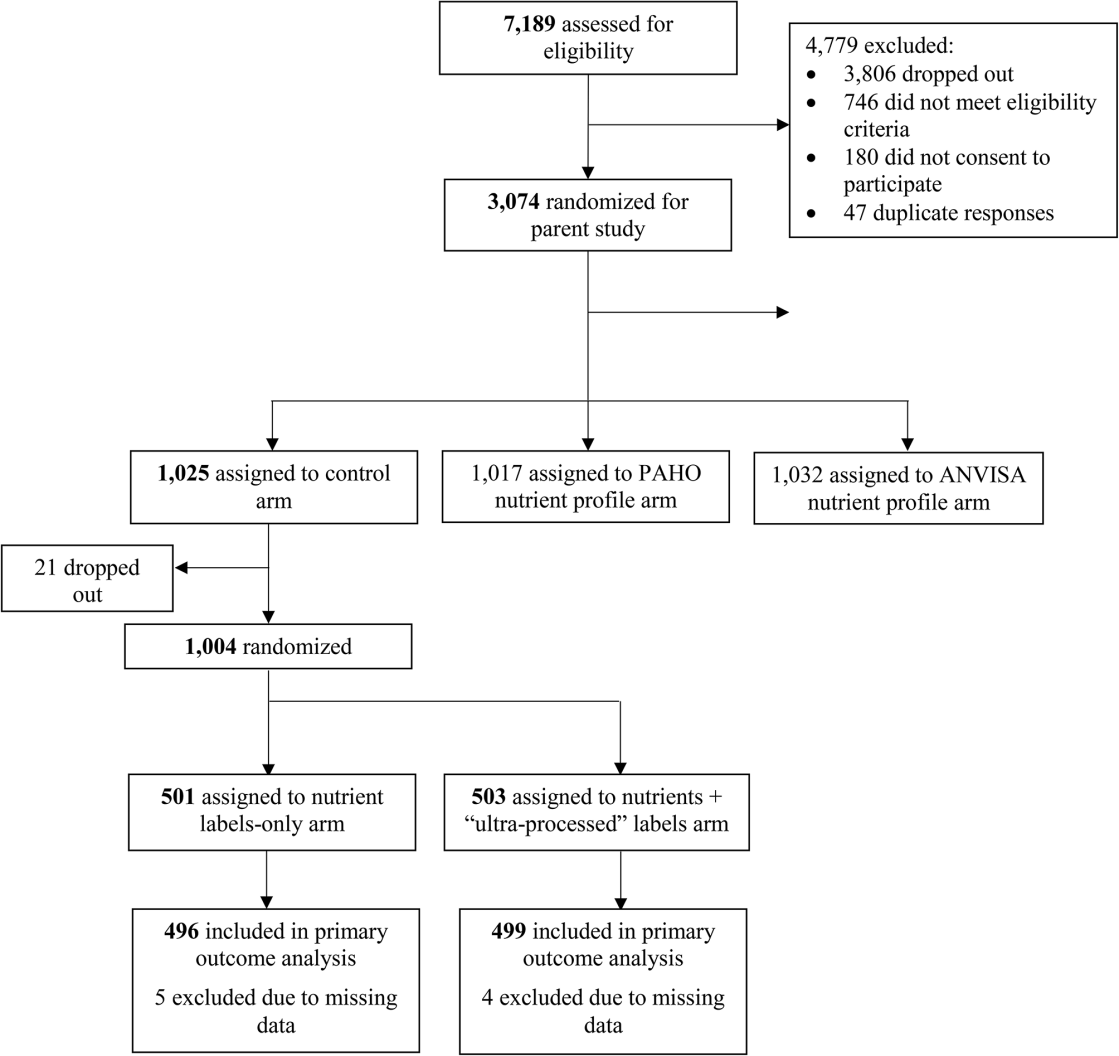
Participant flow diagram

All participants provided free and informed consent. The parent study comprised a shopping task in a fictional online supermarket, an emerging platform for conducting nutrition research [36, 37], followed by a self-administered online survey programmed into Qualtrics survey software. The current study was embedded into the survey. Both studies were conducted entirely in Brazilian Portuguese. To avoid contamination, only participants originally assigned to the parent study’s control group (in which products did not carry any FOPLs) participated in the present study. This procedure resulted in 1,004 participants in the current study (Fig. 1).

**Fig. 2:**
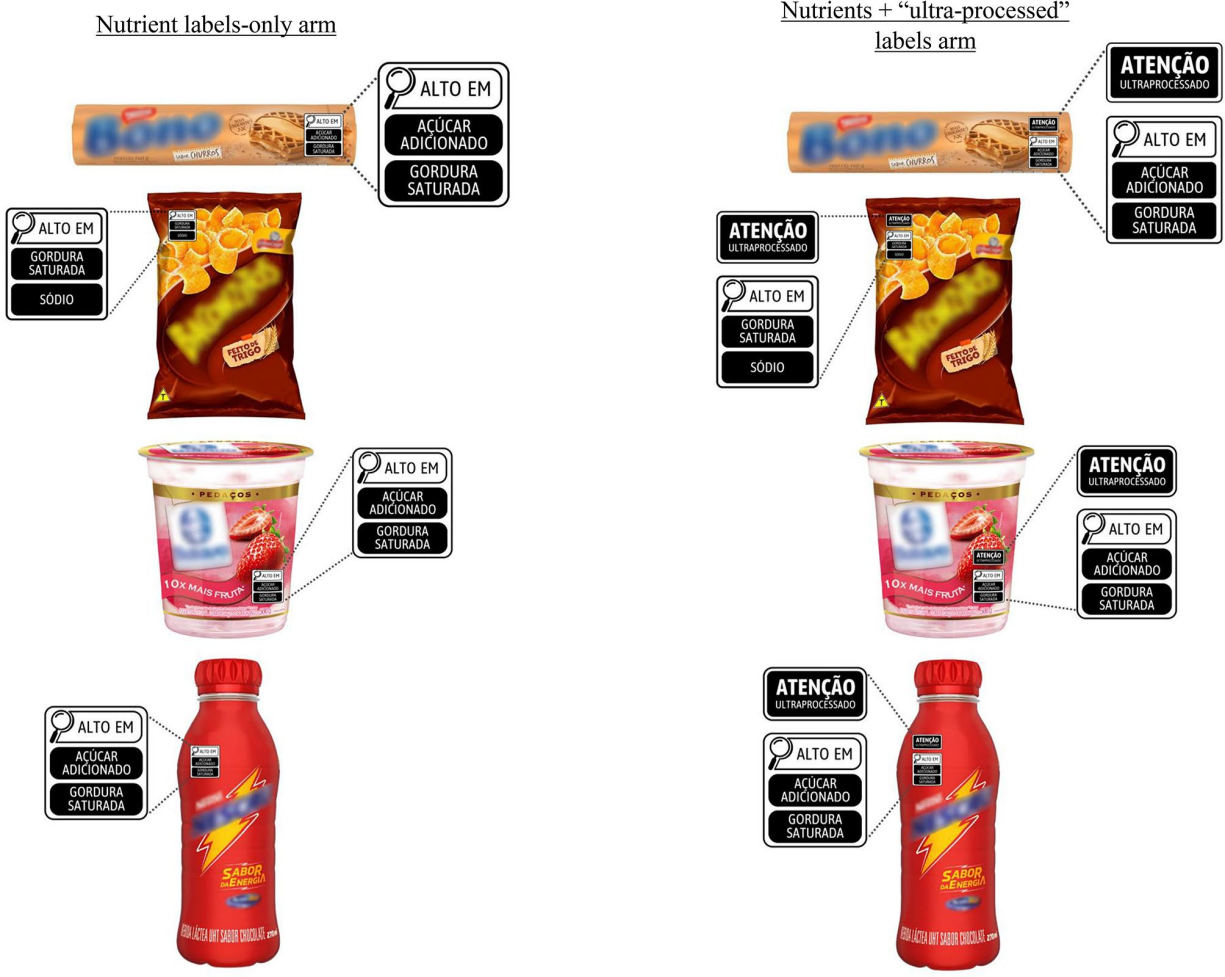
Study stimuli

Participants were randomly assigned to one of two labeling conditions using the Qualtrics randomizer function: nutrients-only labels, in which products carried only Brazil’s current nutrient warning labels, or nutrients plus ultra-processed labels, in which each product carried the same nutrient warning labels and all products carried an additional label that read “WARNING: ULTRA-PROCESSED.” Since the ultra-processed warning label is novel to consumers, we chose to use the marker word “warning” to make this label more attention-grabbing [38] while still generally adhering to Brazil’s FOPL visual identity (i.e., color scheme and shapes). Participants saw images of four products in random order carrying their assigned labels: cookies, salty snacks, a yogurt, and flavored milk (Fig. 2). Cookies and salty snacks are among the UPFs that most contribute to energy intake in Brazil [27, 39]. Yogurt and flavored milk are also widely consumed in Brazil [40], and despite often being ultra-processed and high in sugar, tend to be perceived as healthier in studies from several countries [40–44]. To enhance realism, our stimuli displayed real products from established brands in Brazil that had been available in the online supermarket used in the parent study.

### Measures

At the start of the survey, participants answered the open-ended question “What do you think the term ‘ultra-processed’ means?” and rated their confidence in their understanding of the term “ultra-processed” on a 5-point scale. These measures were placed before the experiment to capture participants’ prior familiarity with the concept of UPFs without the influence of ultra-processed warning labels on products. Participants then proceeded to answer 30 additional questions for the parent study about their experience in the online store and products displayed in the store, which were placed before the experiment as a wash-out.

Next, after seeing each product, participants rated their intentions to buy the products (“How likely would you be to buy this product in the next week, if it were available in a store?”) as the primary outcome. As secondary outcomes, participants identified whether they believed the products to be ultra-processed or not (“Do you think this product is ultra-processed?”), rated the products’ perceived healthfulness (“How healthy does this product seem to you?”), and rated the perceived message effectiveness (PME) of the warning labels with three items: “How much do these labels discourage you from wanting to consume this product?”, “How much do these labels make you concerned about the health effects of consuming this product?”, and “How much do these labels make consuming this product seem unpleasant to you?”. PME is a measure commonly used in message-development studies and has been shown to predict actual message effectiveness [45–48]. Response options to all outcomes except UPF identification were provided on a Likert-style scale ranging from the lowest value (coded as 1) to the highest value (coded as 5); response options to the UPF identification outcome were coded dichotomously (i.e., “no” or “I’m not sure” vs. “yes”). Lastly, the survey also collected information on demographic characteristics.

### Analysis

First, we developed a codebook to code the open-ended responses. This code-development process used a hybrid inductive-deductive approach [49–51] while reviewing around 25% of the responses, guided both by the 2015 Dietary Guidelines for the Brazilian Population’s definition of UPFs [26] and by insights obtained from the data. We captured descriptors used by participants and matched such descriptors to their corresponding UPF or non-UPF characteristics (Table 1). Once the codebook was developed, two Portuguese-speaking coders independently coded all responses and resolved any discrepancies by consensus.

**Table 1:** Descriptors extracted from open-ended responses to “What do you think the term “ultra-processed” means?” (n = 1,004)

Characteristic	Descriptors mentioned in response^†^	*n* ^†^	%^†^
Lack of whole foods	*Processing* • Very/heavily processed • Industrially/chemically/machine processed • Transformed, altered, modified, manipulated, refined, treated • Many processing stages or phases • Processing methods such as blending, grinding, crushing, pre-cooking	567	56%
	*Loss of original foods* • Lack or loss of original/natural food, “base,” or “material” • Substances derived from whole foods or “food-like” • Product made of “leftovers” or “remade”	32	3%
Synthetic substances	*Additives* • Additives, preservatives, colors, aromas, emulsifiers, thickeners, flavorings • Chemicals, unnatural/artificial/lab-made/industrial ingredients, unfamiliar ingredients/not used in home cooking	141	14%
	*Unnaturalness* • Unnatural, synthetic, artificial product	44	4%
Unhealthy products	*Nutrient content* • Excessive nutrients of concern (i.e., calories, fat/oils, sugar, sodium/salt) • Nutritionally poor or unbalanced • Lacking beneficial nutrients (i.e., vitamins, minerals, fiber)	63	6%
	*Unhealthfulness* • Unhealthy or bad/harmful/dangerous to health • Foods that should be avoided for health reasons	70	7%
Other UPF characteristics	*Large number of ingredients* • Large number of ingredients or more than 5 ingredients • Mixing of many foods or ingredients	48	5%
	*Convenience* • Ready-to-eat, ready-to-heat, pre-cooked/fried/prepared, frozen • Fast, practical, efficient, convenient, portable, easy to prepare	57	6%
Non-UPF characteristics	*Food-related but not UPF characteristic* • Food characteristics that are not part of UPF definition (e.g., GMOs, non-organic foods, sanitized/sterilized foods, canned foods, foods containing seasonings/condiments, fermented foods, foods containing hormones, vacuum-sealed foods, non-fresh foods) • Example of UPF used as a definition rather than as example (e.g., equating UPFs to processed meats and providing no other definition) • Foods that are re-sold/put back on the shelf • Any other food-related interpretation	44	4%
	*Positive interpretation* • Assessment of product quality as good • Well prepared or sophisticated product	36	4%
	*Outdated/Surpassed (“* *Ultrapassado* *”)* • Old, outdated, no longer used, out of season, beyond limit, overcome limit, beyond necessary	47	5%
	*Non-food related* • Response does not seem to relate to foods/beverages in any clear way • Response does not seem to make sense in the context of the question • Response refers to processes/processing but is phrased in a way that does not seem to refer to foods/beverages	58	6%
	*Doesn’t know or didn’t answer* • Response states that participant does not know or is unsure what UPFs are • Response states “nothing” (i.e., the term ultraprocessed means nothing to the participant) • Participant did not answer the question (missing response)	49	5%

^†^Characteristics and descriptors are *not* mutually exclusive

*Note*. UPF=ultra-processed food.

To gauge the level of understanding of the UPF concept in the sample, we classified all participants based on what descriptors they mentioned in their responses (Table 2). Because the Dietary Guidelines for the Brazilian Population define UPFs as “industrial formulations made entirely or mostly from substances extracted from whole foods, derived from food constituents, or synthesized in laboratories,” [26] we considered lack of whole foods and presence of synthetic substances as the two key UPF characteristics in this analysis. Participants who mentioned descriptors related to both key UPF characteristics (i.e., at least one lack of whole foods descriptor and at least one synthetic substances descriptor) were thus classified as having a high understanding of the concept. Next, we classified those who mentioned at least one non-UPF characteristic as having a low understanding of the concept, unless they had also mentioned the two key UPF characteristics. Lastly, we verified that all other participants had mentioned at least one descriptor related to a UPF characteristic (i.e., lack of whole foods, synthetic substances, unhealthy products, or other UPF characteristics) and proceeded to classify them as having a moderate understanding of the concept.

**Table 2:** Understanding of term “ultra-processed” extracted from open-ended responses to “What do you think the term “ultra-processed” means?” (*n* = 1,004)

Level of understanding	Characteristics mentioned in response	*n*	%
High understanding	Mentions *key* UPF characteristics as specified by the Dietary Guidelines for the Brazilian Population i.e.,* lack of whole foods AND synthetic substances*	84	8%
Low understanding	Mentions at least one non-UPF characteristic and *not* classified as high understanding *i.e. non-UPF characteristic NOT (lack of whole foods AND synthetic substances)*	232	23%
Moderate understanding	Mentions at least one UPF characteristic and *not* classified as high understanding or low understanding i.e.,* lack of whole foods OR synthetic substances OR unhealthy products OR other UPF characteristics NOT (lack of whole foods AND synthetic substances) NOT non-UPF characteristic*	688	69%

Note. UPF = ultra-processed food

For the experimental portion of this study, given our pre-determined sample size of around 1,000, 80% power, and a critical alpha of 0.05, we calculated that we would be able to detect a minimum effect size of d = 0.15 for the primary outcome (i.e., purchase intentions). We determined that this sample size would be sufficient, given that, on a smaller preliminary study using ultra-processed warning labels, we found an effect size of d = 0.22 on a single-item PME measure [34].

For continuous outcomes (i.e., purchase intentions, perceived healthfulness, PME), we verified that reliability across products was sufficient (Cronbach’s α > 0.7, Table S1) and averaged participants’ responses to each question across the four products. For PME, the only outcome measured through more than one item (Cronbach’s α = 0.92), we also averaged participants’ responses across items. We conducted independent samples t-tests to examine the statistical significance of the differences between labeling conditions. For the identification of UPFs outcome, we created a dichotomous variable indicating whether each participant correctly identified all four products as ultra-processed or not, and then conducted a chi-squared test for significance testing. Additionally, for each of these outcomes, we conducted exploratory analyses (not included in the prospective trial registration) to determine if there were differences between labeling conditions for each individual product. These exploratory analyses used the same statistical testing approach as our primary analyses – i.e., independent samples t-tests for continuous outcomes and chi-squared tests for the dichotomous outcome.

We also conducted moderation analyses to examine whether demographic characteristics (i.e., age, gender, educational attainment, self-reported health status), self-reported confidence in one’s understanding of the term “ultra-processed,” and low understanding of the term “ultra-processed” measured through the open-ended response moderated the effect of the ultra-processed warning label on identification of UPFs and purchase intentions. All moderation analyses were included in the prospective trial registration except for moderation by self-reported confidence in understanding of the term “ultra-processed,” which we added post-hoc to provide a more comprehensive understanding of how results may have differed among participants. We fit a series of logistic regressions for the dichotomous outcome (i.e., UPF identification) and linear regressions for the continuous outcome (i.e., purchase intentions), with separate models for each potential moderator. Each model included the trial arm, the moderator, and their interaction. Moderator variables were dichotomized to maximize statistical power due to the limited number of participants in certain response categories, as well as to simplify interpretation of results. Additionally, to account for multiple comparisons, we performed the Holm-Bonferroni correction on the p-values associated with the interaction terms.

We used complete case analysis to address any missing data, resulting in the exclusion of 7 participants from the analysis of UPF identification, 9 participants from purchase intentions, 8 participants from perceived healthfulness, and 18 participants from PME. Analyses were conducted using G*Power3.1 (power calculations) and Stata/SE version 17 (all other analyses) with a critical alpha of 0.05.

## Results

Participants’ mean age was 37.1 years (SD = 12.2) and 54% identified as men. The sample had a high level of educational attainment compared to the Brazilian population: around 62% of participants had a bachelor’s degree or more, compared to 19% of the Brazilian population [52]. Around 53% of participants identified white, 33% as *pardo* (i.e., “brown” or mixed race), and 12% as Black – compared to 44%, 45%, and 10%, respectively, of the Brazilian population [53]. In terms of regional distribution, the sample resembled the Brazilian population: 46% of participants reported living in the Southeast, 30% in the Northeast, and 12% in the South – compared to 42%, 27%, and 15%, respectively, of the population [53]. More than half of participants reported that they were confident in their ability to identify ultra-processed foods prior to the experiment (Table 3).

**Table 3:** Participant characteristics (*n* = 1,004)

Characteristics	*n*	%
Gender		
Man	538	54%
Woman	461	46%
Other	5	< 1%
**Age**		
18–29	318	32%
30–39	289	29%
40–54	292	29%
≥55	105	10%
**Region**		
Southeast	463	46%
Northeast	297	30%
South	121	12%
Center-West	60	6%
North	63	6%
**Education** (n = 995)		
Less than high school	15	2%
High school or some college	360	36%
Higher education	620	62%
**Race** (n = 994)		
White	523	53%
Black	121	12%
Asian	23	2%
Brown	324	33%
Indigenous	3	< 1%
Monthly household income* (n = 995)		
Up to 2,200 BRL	157	16%
2,201-5,500 BRL	359	36%
5,501 − 11,000 BRL	289	29%
11,101 − 22,000 BRL	130	13%
More than 22,000 BRL	60	6%
**Confidence in UPF understanding**		
Not confident or indifferent	423	42%
Confident	581	58%
**Self-reported health status** (n = 995)		
Poor or very poor	71	7%
Fair, good, or very good	924	93%
**Self-reported diabetes** (n = 995)	85	9%
**Self-reported hypertension** (n = 995)	156	16%
**Self-reported heart disease** (n = 995)	34	3%

*As of June 1st 2023, 1 USD = 5 BRL; the mean per capita household income in Brazil in 2023 was 1,893 BRL

*Note*. UPF=ultra-processed food

Table 1 details the descriptors that participants used in their open-ended responses related to meaning of the term “ultra-processed.” The most used descriptor, employed by 56% of participants, was a reference to food processing, either in a tautological manner (e.g., “very processed”) or using other terms to describe food transformation processes. The second most used descriptor, employed by 14% of participants, was a reference to additives, either directly or using terms that allude to additives, such as “artificial ingredients,” “lab-made ingredients,” and “ingredients not used in home cooking.” Overall, 58% of participants used at least one descriptor that referred to lack of whole foods as a UPF characteristic, and 18% used at least one descriptor that referred to synthetic substances. Other descriptors used by smaller percentages of participants included nutrient content (6%), large number of ingredients (5%), and convenience (6%). Around 7% of participants used unhealthfulness as a UPF descriptor.

Participants also used descriptors that did not refer to any UPF characteristics. Around 4% of participants mentioned descriptors that are food-related but not specifically UPF-related, such as “genetically modified foods,” “non-organic foods,” or “non-fresh foods,” among others. Around 5% provided answers that defined UPFs as “outdated” or “surpassed” – possibly a result of misreading the Portuguese word for ultra-processed (*ultraprocessado*) as a similar word with this meaning (*ultrapassado*). Around 4% provided answers that exhibited a positive interpretation of UPFs as “good-quality” or “sophisticated” products, which contradicts the Brazilian Dietary Guidelines’ description of UPFs. Lastly, some participants provided non-food related descriptors (6%), stated that they did not know what the term “ultra-processed” means, or did not provide a response (5%). Based on their use of these descriptors, we classified 8% of the sample as having a high understanding, 69% as having a moderate understanding, and 23% as having a low understanding of the concept of UPF (Table 2).

**Fig. 3:**
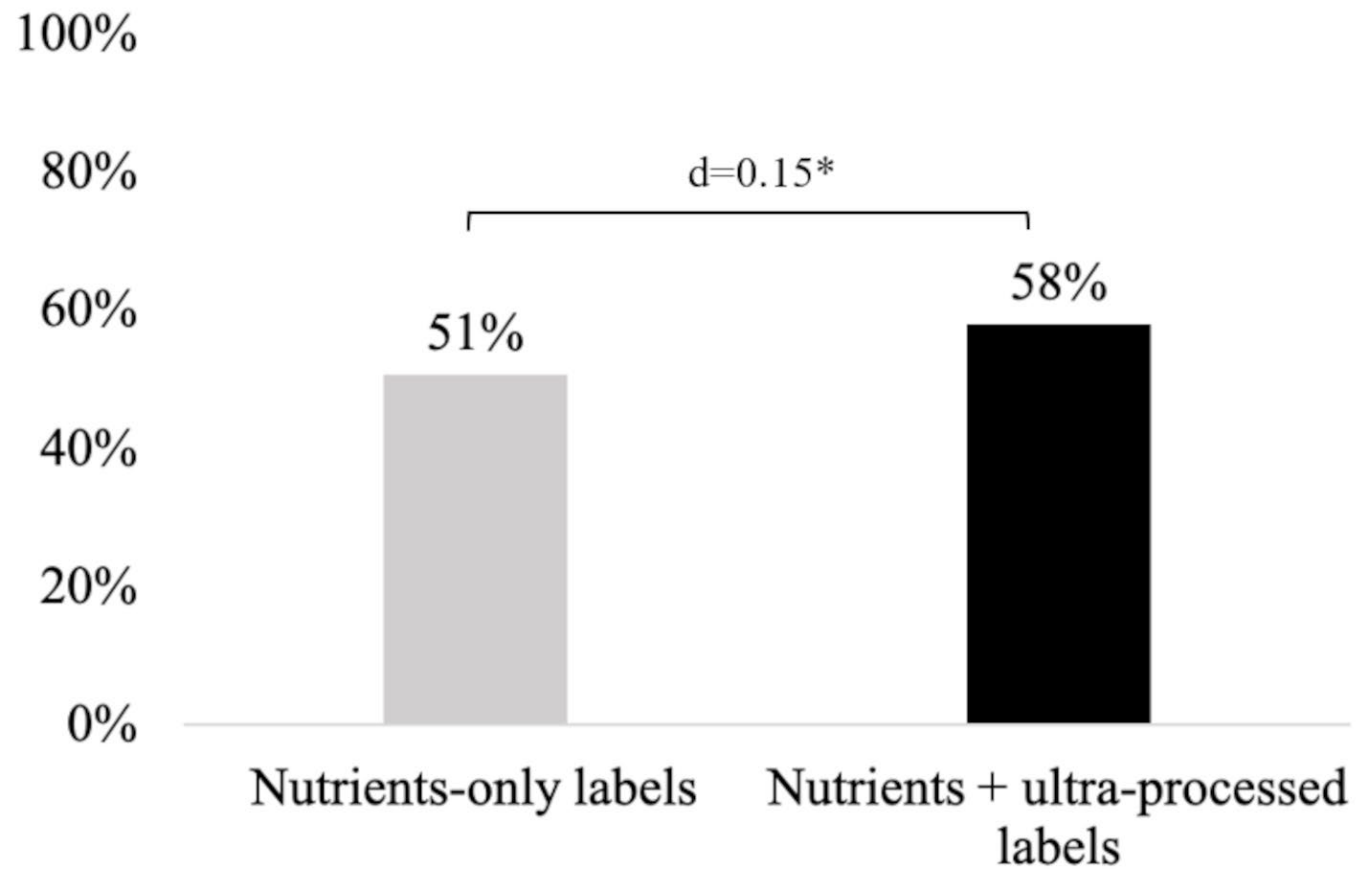
Percentage of participants who correctly classified all products as ultra-processed by study arm (*n* = 997) *Statistically significant difference at the 95% confidence level *Note.* d = Cohen’s d

**Fig. 4:**
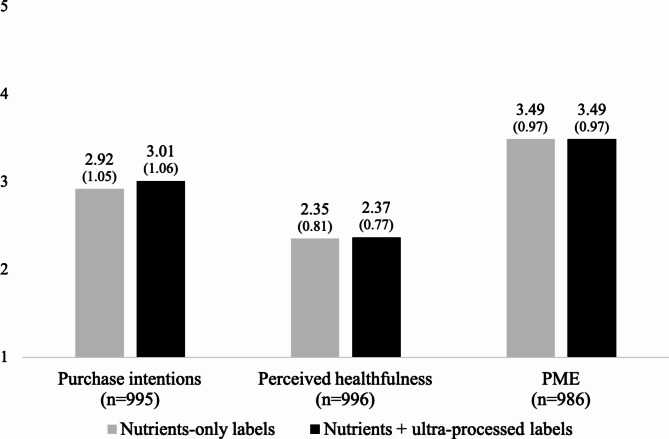
Product purchase intentions, product perceived healthfulness, and perceived message effectiveness of labels by study arm (means and standard deviations) *Note.* PME = perceived message effectiveness *Note.* Response options ranging from low (1) to high (5) values

The experiment demonstrated that a higher share of participants who saw the nutrients plus ultra-processed warning labels correctly identified all products shown as being UPFs, compared to participants who saw nutrients-only warning labels (58% vs. 51% respectively, d = 0.15, *p* = 0.021, Fig. 3). However, the nutrients plus ultra-processed warning labels did not differ from nutrients-only warning labels on participants’ intentions to purchase the products, perceived product healthfulness, and perceived label effectiveness (all *p* > 0.05, Fig. 4). Similarly, there were no differences by experimental arm on purchase intentions, perceived product healthfulness, and perceived label effectiveness when examining effects for individual products (Table S1).

Lastly, moderation analyses did not reveal significantly different effects of experimental arm on identification of products as ultra-processed or purchase intentions by age, gender, education, self-reported health status, self-reported confidence in understanding of the term “ultra-processed,” or low understanding of the term “ultra-processed” (all *p* > 0.05, Tables S2 and S3).

## Discussion

In this online study with Brazilian adults, we experimentally examined whether adding a label warning consumers that a product is ultra-processed to Brazil’s existing nutrient warning labels influenced participants’ ability to identify the product as ultra-processed, their product perceptions, and behavioral intentions. The addition of the ultra-processed warning label led to a higher share of participants being able to identify UPFs compared to nutrient warning labels alone. On the other hand, the addition of the ultra-processed warning label did not influence participants’ intentions to purchase the products, how healthful they perceived the products to be, or how effective they perceived the labels to be. To contextualize our experimental findings, we also qualitatively explored underlying understandings of the meaning of the term “ultra-processed” in the sample. Most participants exhibited a moderate understanding of the UPF concept.

Our results suggest that front-of-package ultra-processed warning labels could help consumers more easily identify which products are UPFs, which is a key initial step toward better-informed consumer choices. The moderate understanding of the term “ultra-processed” observed in our sample underscores the importance of providing consumers with this sort of practical guidance for navigating a food supply abundant in UPFs. Along similar lines, previous studies in Brazil and other Latin American countries have shown that, although consumers can sometimes distinguish between unprocessed and ultra-processed foods, most have difficulty with foods that fall in intermediate categories (e.g., processed culinary ingredients and processed foods) [54–56], which are an important part of and facilitate healthy dietary patterns. However, it is worth noting that although the presence of the ultra-processed warning label improved participants’ ability to identify UPFs, 42% still failed at this identification, suggesting that complementary strategies such as public health campaigns may also be necessary to improve consumer literacy around UPFs. Additionally, our sample had a high level of educational attainment compared to the general Brazilian population, so it would be important determine whether ultra-processed warning labels would also help consumers with lower educational attainment identify UPFs.

On the other hand, our finding that ultra-processed warning labels did not influence participants’ product perceptions or behavioral intentions differs from previous studies on ultra-processed food labels, which observed effects on these outcomes when comparing ultra-processed labels to control [34, 35]. This difference suggests that, although ultra-processed labels may have an influence on consumers when compared to the absence of any FOPLs, their influence may not go beyond the influence already exerted by nutrient labels. A possible explanation may be that the difference between nutrient content and degree of processing is not evident to consumers, in which case the added ultra-processed warning label may have seemed redundant. While there is currently no scientific consensus about the importance of this distinction, emerging epidemiological evidence suggests that level of processing may matter for health independently from nutrient content [18–24] – therefore, future health communication research should examine whether results would differ when making this distinction clearer. Additionally, different UPF labeling approaches would be also worth exploring in future research. One such approach recently proposed in the United States Senate are health warning labels – i.e., labels warning consumers about the possible health effects of UPF consumption rather than simply identifying a product as ultra-processed [57]. Another approach would be signaling specific non-nutrient related UPF characteristics, such as the presence of food additives, which may be more easily understood or more effectively impact consumers’ health risk perceptions than the broader and more complex term “ultra-processed.” An example of this approach can be found in Mexico and Colombia’s FOPL systems, which currently signal the presence of non-sugar sweeteners [58].

Lastly, it is crucial to consider that, in countries adopting nutritional warning label schemes like Brazil and other nations in the Americas, incorporating ultra-processed warning labels could result in many products that do not display nutrient warnings (because they do not exceed pre-determined amounts of nutrients of concern) being obligated to carry ultra-processed warnings. For instance, a study analyzing the 80 products that constitute a food basket in Chile found that 33% those classified as UPFs based on the Nova system did not carry any nutrient warnings [59]. Similarly, a recent study examining a nationally representative sample of packaged foods purchased in the US found that a Nova-based approach would target 50% of products for intervention, while an exclusively nutrient-based approach would only target 43% of products and thus miss an important share of UPFs [60]. Recent studies also indicate that, after the implementation of nutrient-based FOPLs, manufacturers tend to reformulate several products to avoid mandatory labels – and while such reformulations generally improve the nutrient content of products, they often result in manufacturers incorporating additives, such as non-sugar sweeteners, that can categorize products as UPFs [61–63]. To maximize power with our pre-determined sample size, this study did not examine the potential benefits offered by ultra-processed warning labels in these scenarios, which merit further investigation.

This study’s strengths include an experimental design allowing for causal inference, the use of professionally developed stimuli featuring multiple types of real products, and a diverse national sample. However, this study also has limitations. As an online experiment with a convenience sample, we cannot establish how generalizable our findings would be in real-world settings – and although previous evidence suggests that online convenience samples tend to produce experimental results similar in direction to nationally representative samples [64, 65], our sample’s small share of participants with low educational attainment may have specifically precluded us from findings differences in the effectiveness of ultra-processed warning labels by education. The experiment exposed participants to only four products, so we cannot establish how generalizable our findings would be to a broader set of products. Additionally, given a lack of applicable measures validated for Brazilian samples specifically, we relied on measures validated in samples from other countries and commonly used in food labeling studies around the world [45–48, 66]. The study also did not inform participants about the meaning and intended use of ultra-processed warning labels prior to label exposure, which could influence effectiveness in a real-world implementation scenario. Lastly, to increase realism, we designed rectangular ultra-processed labels that would be compatible with and proportional in size to Brazil’s current FOPL visual identity, but alternative design elements such as octagonal or triangular shape [38, 67–72] and a larger size could possibly heighten the effectiveness of ultra-processed warning labels.

## Conclusion

Existing evidence indicates that front-of-package labels can be an effective strategy to reduce purchases of unhealthful products. Our study suggests that adding ultra-processed warning labels to nutrient warning labels may help consumers more easily identify whether a product is ultra-processed, but may not influence consumers’ product perceptions and behavioral intentions beyond the influence already exerted by nutrient labels. Future research should examine the potential effects of ultra-processed warning labels when applied to products that do not require nutrient labels, as well as the potential benefits of alternative labeling approaches signaling possible health effects of UPF consumption or specific UPF characteristics.

## Electronic supplementary material

Below is the link to the electronic supplementary material.

Supplementary Material 1

Supplementary Material 2

Supplementary Material 3

## Data Availability

The dataset supporting the conclusions of this article is available in the Open Science Framework repository [https://osf.io/6na8q/?view_only=48c9e27e72f746ecba0a3970675a10b3].

## Abbreviations

UPF: Ultra-processe food
FOPL: Front-of-package labeling/labels
PME: Perceived message effectiveness
