# Supplementary material for: “Warning: ultra-processed”: an online experiment examining the impact of ultra-processed warning labels on consumers’ product perceptions and behavioral intentions
Source: Int J Behav Nutr Phys Act. 2024 Oct 9;21:115. doi: 10.1186/s12966-024-01664-w (PMC11462959; doi:10.1186/s12966-024-01664-w)
Supplement: Supplementary file 2 — Supplementary Material 2 [file 12966_2024_1664_MOESM2_ESM.docx]

**Notes on Sample for Empirical Study**

**Sample recruitment:** We recruited an online sample using the survey research platform Cint. Participants were eligible if they were 18 years or older, resided in Brazil, and were responsible for at least 50% of their household’s food purchases.

**Sample representativeness:** We recruited a convenience sample that was not representative of and had a higher level of educational attainment compared to the Brazilian population. We acknowledge this as a limitation in our manuscript. However, the panel company used purposive sampling to obtain a sample whose age, gender, and regional distribution was comparable to that of the Brazilian population. It is also worth noting that previous evidence suggests that online convenience samples tend to produce experimental results similar in direction to nationally representative samples.

**Missing data and how the analyzed sample differed from the recruited sample:** Per our registered analytic plan, we used complete case analysis to address any missing data – i.e., we excluded participants from the analysis if they did not provide a response to an outcome measure for any of the 4 products shown during the experiment. This resulted in the exclusion of 7 participants from the analysis of UPF identification, 9 participants from purchase intentions, 8 participants from perceived healthfulness, and 18 participants from perceived message effectiveness.
