# Supplementary material for: “Warning: ultra-processed”: an online experiment examining the impact of ultra-processed warning labels on consumers’ product perceptions and behavioral intentions
Source: Int J Behav Nutr Phys Act. 2024 Oct 9;21:115. doi: 10.1186/s12966-024-01664-w (PMC11462959; doi:10.1186/s12966-024-01664-w)
Supplement: Supplementary file 3 — Supplementary Material 3 [file 12966_2024_1664_MOESM3_ESM.docx]

**Table S1. Mean (SD) purchase intentions, perceived healthfulness, perceived message effectiveness, and percentage of participants who identified ultraprocessed products, pooled and by product**

|  |  | **Nutrients-only labels (n=501)** | | **Nutrients+ultra-processed labels (n=503)** | | | | **Cronbach’s**  **alpha** | **p-value** |
| --- | --- | --- | --- | --- | --- | --- | --- | --- | --- |
|  |  | Mean | SD |  | Mean | SD |  |  |  |
| **Purchase intentions** (all products) | | 2.92 | 1.06 |  | 3.01 | 1.06 |  |  | 0.19 |
|  | Cookies | 2.86 | 1.23 |  | 2.93 | 1.27 |  | 0.86 | 0.39 |
|  | Salty snack | 2.69 | 1.29 |  | 2.78 | 1.33 |  |  | 0.26 |
|  | Chocolate milk | 2.99 | 1.26 |  | 3.06 | 1.30 |  |  | 0.41 |
|  | Yogurt | 3.14 | 1.20 |  | 3.27 | 1.20 |  |  | 0.09 |
| **Perceived healthfulness** (all products) | | 2.35 | 0.81 |  | 2.37 | 0.78 |  |  | 0.76 |
|  | Cookies | 2.18 | 0.89 |  | 2.17 | 0.88 |  | 0.86 | 0.89 |
|  | Salty snack | 1.92 | 0.91 |  | 1.92 | 0.89 |  |  | 0.97 |
|  | Chocolate milk | 2.52 | 0.99 |  | 2.55 | 0.99 |  |  | 0.58 |
|  | Yogurt | 2.80 | 1.01 |  | 2.83 | 1.00 |  |  | 0.62 |
| **Perceived message effectiveness** (all products) | | 3.49 | 0.89 |  | 3.49 | 0.92 |  |  | 0.96 |
|  | Cookies | 3.55 | 0.94 |  | 3.54 | 1.01 |  | 0.93 | 0.88 |
|  | Salty snack | 3.65 | 1.00 |  | 3.65 | 1.02 |  |  | 0.94 |
|  | Chocolate milk | 3.43 | 0.97 |  | 3.43 | 1.03 |  |  | 0.91 |
|  | Yogurt | 3.35 | 0.96 |  | 3.36 | 0.99 |  |  | 0.93 |
|  |  | % | | % | | | |  |  |
| **Correct UPF identification** (all products) | | 51% | | 58% | | | |  | **0.021** |
|  | Cookies | 74% | | 84% | | | |  | **<0.001** |
|  | Salty snack | 80% | | 86% | | | |  | **0.011** |
|  | Chocolate milk | 69% | | 75% | | | |  | **0.023** |
|  | Yogurt | 67% | | 72% | | | |  | **0.055** |

*Note.* UPF=ultra-processed food

**Table S2. Marginal effect of nutrients plus ultra-processed labels on purchase intentions compared to nutrients-only labels, by participant characteristics**

|  | | **β** | **95% CI** | **Holm-Bonferroni adjusted p-value for interaction** |
| --- | --- | --- | --- | --- |
| **Age** | |  |  | 1.00 |
|  | 18-39 | 0.09 | -0.07,0.26 |  |
|  | 40+ | 0.04 | -0.16,0.25 |  |
| **Gender** | |  |  | 1.00 |
|  | Female or other | 0.07 | -0.12,0.26 |  |
|  | Male | 0.13 | -0.05,0.31 |  |
| **Education** | |  |  | 0.91 |
|  | High school or less | 0.06 | -0.24,0.35 |  |
|  | College or more | 0.08 | -0.07,0.22 |  |
| **Health status** | |  |  | 1.00 |
|  | Poor or very poor | 0.13 | -0.36,0.62 |  |
|  | Fair, good or very good | 0.08 | -0.05,0.22 |  |
| **Confidence** | |  |  | 1.00 |
|  | Not confident or indifferent | 0.06 | -0.15,0.26 |  |
|  | Confident | 0.11 | -0.06,0.29 |  |
| **Understanding** | |  |  | 1.00 |
|  | High or moderate | 0.06 | -0.09,0.20 |  |
|  | Low | 0.16 | -0.10,0.43 |  |

**Table S3. Marginal effect of nutrients plus ultra-processed labels label on probability of UPF identification compared to nutrients-only labels, by participant characteristics**

|  |  | **ΔPr(Y)** | **95% CI** | **Holm-Bonferroni adjusted p-value for interaction** |
| --- | --- | --- | --- | --- |
| **Age** | |  |  | 1.00 |
|  | 18-39 | 0.06 | -0.02,0.14 |  |
|  | 40+ | 0.09 | >-0.01, 0.19 |  |
| **Gender** | |  |  | 0.72 |
|  | Female or other | 0.01 | -0.08,0.10 |  |
|  | Male | 0.11 | 0.03,0.20 |  |
| **Education** | |  |  | 1.00 |
|  | High school or less | 0.05 | -0.09,0.18 |  |
|  | College or more | 0.09 | 0.02,0.16 |  |
| **Health status** | |  |  | 1.00 |
|  | Poor or very poor | -0.07 | -0.30,0.16 |  |
|  | Fair, good or very good | 0.09 | 0.02,0.15 |  |
| **Confidence** | |  |  | 0.80 |
|  | Not confident or indifferent | 0.06 | -0.03,0.16 |  |
|  | Confident | 0.08 | >-0.01,0.16 |  |
| **Understanding** | |  |  | 1.00 |
|  | High or regular | 0.09 | 0.02,0.16 |  |
|  | Low | 0.04 | -0.08,0.17 |  |

*Note.* UPF=ultra-processed food
